# Medical Reform

**Published:** 1846-03

**Authors:** 


### MEDICAL EXAMINER,

AND

### RECORD OF MEDICAL SCIENCE.

NEW SERIES.—No. XV.—MARCH, 1846.

---

### ORIGINAL COMMUNICATIONS.

[The following communication relates to a subject of great im portance, especially at the present time. The author is one who loves his profession, and feels a deep interest in all that concerns its honor and usefulness; and whatever may be thought of the practicability and expediency of his scheme, all must admit that some improvement in the present state of things is desirable. Whether it is possible to carry reform to the extent proposed by our correspondent, in our present political and social condition, may well be doubted; the subject, however, is one eminently deserving of consideration.]

### MEDICAL REFORM.

At this moment when there are in Philadelphia four chartered institutions, and two voluntary associations, in all of which complete courses of medical Lectures are, or will be, delivered; when we have reason to believe that arrangements are in progress for enlarging still further the corps of public medical instructors; and when, moreover, the number of students resorting to this city appears to be rapidly increasing;—ought not those who stand at the head of the profession to consider the time peculiarly opportune for making the improvements in medical education which all have long admitted to be useful, but none have yet had the resolution to adopt? The present state of things cannot be followed by the ancient *statu quo;* there must be a movement in in one direction or the other. The existing competition in teaching must result either in the subsidence of all the schools to a level with the lowest, or in the extension and elevation of some one among them to such a degree as to defy all rivalry, and such as will furnish the student with an education fitted to keep him up with the march of medical science. As sure as fate a change must come. Let us hope that it will be commenced voluntarily, and with a good grace, by the institutions that have hitherto held an admitted and deserved pre-eminence. Let them not wait till it is thrust upon them, until their foundations are upheaved by the pressure from below, until the glory of reform is obscured by the necessity of submitting to it. They are strong enough in reputation, ability and virtue, to remodel their systems without impairing them, and to impose their own laws upon inferior rivals under the penalty of dissolution for non-obedience. Whether that example be followed or not, the student and the medical profession will be the gainers, and their own fame will be enhanced.

But what improvements should be made? These:—and whether adopted this year, or the next, or still later, beyond all peradventure there are those now in the profession who will live to see them all accomplished.

1st. There should be an examination of every student preliminary to matriculation, in which he should prove his acquaintance with the English language, or his native tongue, with the principal mechanical laws, and with the elements of inorganic chemistry.

2d. The annual term of Lectures should be extended to nine months, and not more than four lectures should be delivered on any one day.

3d. There should be a Professorship of Pathological Anatomy. The Lecturer on Chemistry should confine his attention to pharmaceutical and organic Chemistry. The Lecturer on Obstetrics should deliver a full course on the diseases of women and children. The Lecturers on Medicine and Surgery should treat of General Pathology in its application to their respective departments.

4th. There should be ample provision made for clinical (not amphitheatrical) instruction, but only for students in their second course.

5th. The examination for a degree should be public, and the candidate be obliged to *defend* his Thesis.

6th. No student should be permitted to receive a diploma who has not passed a satisfactory examination on ALL the branches in which he has been instructed.

These propositions are almost self-evident; but if there is any one who denies or doubts them, the writer pledges himself to bring forward such proof of the evils they are intended to cure, as will silence denial and remove every doubt.

Will none of our institutions adopt them? Will not our Professors enter into a Holy Alliance to support one another in carrying them out? If they would preserve for our city its scientific reputation; if they would wither the weeds which infest the country and threaten to overshadow them; if they would be honoured and admired by the present generation, and gratefully remembered by posterity, they will heed this earnest voice, though it be neither the first nor the most emphatic that has sounded the alarm. But if they are deaf to its warning, or timidly refuse to place themselves in the van of the approaching revolution, it requires no prophetic vision to discover that the sentence has gone out against them,—that the *Mene Tekel* is written above their doors. A. S.

